# Expression of Concern: The Gene Expression Analysis of Blood Reveals *S100A11* and *AQP9* as Potential Biomarkers of Infective Endocarditis

**DOI:** 10.1371/journal.pone.0278662

**Published:** 2022-12-13

**Authors:** The PLOS ONE Editors

This article [1] has been identified as one of a series of submissions for which we have concerns about the reported research ethics approval information and the article’s adherence to PLOS research ethics policies.

PLOS will be investigating these concerns in accordance with COPE guidance and journal policies. Meanwhile, the *PLOS ONE* Editors issue this Expression of Concern.
